# Supplementary material for: Mediterranean and MIND Dietary Patterns and Cognitive Performance in Multiple Sclerosis: A Cross-Sectional Analysis of the UK Multiple Sclerosis Register
Source: Nutrients. 2025 Oct 22;17(21):3326. doi: 10.3390/nu17213326 (PMC12608189; doi:10.3390/nu17213326)
Supplement: Supplementary file 1 [file nutrients-17-03326-s001.zip › nutrients-3896917-supplementary.pdf]

Supplementary Table S1. Diet scores calculation

Foods/food groups included in the aMED based on availability in the food frequency questionnaire		
**Food groups**	**Foods**	**Criteria for aMED scoring[1]**
**Vegetables**	Tomatoes, carrots, spinach, broccoli, sprouts, cabbage, peas, green beans, marrow, cauliflower, parsnips, leeks, onions, garlic, mushrooms, sweet petters, beansprouts, green salad, watercress, tomatoes, sweetcorn, beetroot, avocado	1 point for consumption ≥ sex-specific median of study population
**Legumes**	Baked beans, dried lentils, beans, peas, tofu	1 point for consumption ≥ sex-specific median of study population
**Fruit**	Dried fruit & tinned fruit, limited to 1 serve/day; Oranges, apples, pears, banana, melons (cantaloupe, dew melon, watermelon, etc), grapes, strawberries, raspberries, kiwi fruit, peaches, plums, apricots.	1 point for consumption ≥ sex-specific median of study population
**Nuts**	Nuts, peanut butter	1 point for consumption ≥ sex-specific median of study population
**Whole grains**	Brown bread and rolls, whole-meal bread and rolls, brown rice, whole-meal pasta	1 point for consumption ≥ sex-specific median of study population
**Red and processed meat**	Beef, beefburgers, pork, lamb, chicken, bacon, ham, corned beef, sausages, liver	1 point for consumption < sex-specific median of study population[2]
**Fish**	Fried fish, fish fingers/cakes, other white fish, oily fish, shellfish, fish roe	1 point for consumption ≥ sex-specific median of study population
**Ratio of monounsaturated to saturated fat**	Monounsaturated and saturated fat intake	1 point for intakes ≥ sex-specific median of study population
**Ethanol**	Alcohol intake from all sources (wine, beer, port, sherry, spirits)	1 point for intakes within the following range:<br>M, 10-25 g/day<br>F, 5-15 g/day
**Foods/food groups included in the MIND diet based on availability in the food frequency questionnaire**		
**Food Component**	**Examples**	**Recommended Intake for 1 Point**
**Green leafy vegetables**	Spinach, kale, collards, lettuce	≥6 servings/week
**Other vegetables**	Carrots, broccoli, squash, bell peppers	≥1 serving/day
**Nuts**	Almonds, walnuts, peanuts, etc.	≥5 servings/week

**Berries**	Blueberries, strawberries	≥2 servings/week
**Beans**	Lentils, chickpeas, black beans, etc.	≥3 meals/week
**Whole grains**	Brown rice, oats, whole grain bread, whole wheat pasta	≥3 servings/day
**Fish**	Salmon, mackerel, sardines	≥1 meal/week
**Poultry**	Chicken, turkey	≥2 meals/week
**Olive oil**	Olive oil as cooking or dressing oil	Primary oil used at home
**Wine**	Red or white wine	≤1 glass/day
**Red meats**	Beef, pork, lamb, processed meats	<4 servings/week
**Butter & stick margarine**	Solid fats	<1 tablespoon/day
**Cheese**	All types including processed cheese	<1 serving/week
**Pastries & sweets**	Cakes, cookies, candy, ice cream	<5 servings/week
**Fried/fast food**	Fried chicken, fries, takeaways, burgers	<1 serving/week

Note: aMED, alternate Mediterranean die score; F, females; M, males [1]Zero points for not meeting the criteria.

Sensitivity analysis:

Supplementary Table S2. Sensitivity analysis using rank-based inverse normal transformed Deviation from Expected (DfE) scores: associations with aMED and MIND diet scores

Total aMED			Total MIND		
**Cognitive scores**	***β*** **(95%CI)**	**p-value**	**Cognitive scores**	***β*** **(95%CI)**	**p-value**
G: Global	0.03 [-0.04, 0.11]	0.393	G: Global	0.07 [-0.01, 0.14]	0.083
PC0: Objects memory	-0.06 [-0.13, 0.02]	0.118	PC0: Objects memory	-0.00 [-0.08, 0.07]	0.944
PC1: Problem solving	0.03 [-0.04, 0.10]	0.446	PC1: Problem solving	0.05 [-0.02, 0.12]	0.163
**PC2: IPS**	**-0.08 [-0.15, -0.00]**	**0.037**	PC2: IPS	-0.03 [-0.10, 0.05]	0.468
PC3: Words memory	0.03 [-0.04, 0.11]	0.408	**PC3: Words memory**	**0.07 [0.00, 0.14]**	**0.045**
2D Manipulations	-0.02 [-0.10, 0.05]	0.554	2D Manipulations	-0.02 [-0.09, 0.05]	0.579
Blocks	-0.03 [-0.11, 0.04]	0.382	Blocks	0.03 [-0.05, 0.10]	0.518
Card Pairs	-0.02 [-0.10, 0.05]	0.507	Card Pairs	0.03 [-0.04, 0.09]	0.467
Motor Control	-0.06 [-0.14, 0.01]	0.101	Motor Control	-0.02 [-0.10, 0.05]	0.543
Objects Memory Delayed	-0.04 [-0.11, 0.03]	0.271	Objects Memory Delayed	0.02 [-0.05, 0.10]	0.578
Objects Memory Immediate	-0.03 [-0.11, 0.04]	0.405	Objects Memory Immediate	0.00 [-0.08, 0.08]	0.913
**SRT**	**-0.08 [-0.15, -0.00]**	**0.046**	SRT	-0.06 [-0.14, 0.01]	0.101
Switching Stroop	0.05 [-0.03, 0.12]	0.228	Switching Stroop	0.07 [-0.00, 0.15]	0.060
Trail Making	-0.04 [-0.12, 0.03]	0.221	Trail Making	-0.04 [-0.12, 0.03]	0.291
**Verbal Analogies**	**0.09 [0.01, 0.17]**	**0.021**	Verbal Analogies	0.04 [-0.04, 0.11]	0.351
**Word Definitions**	**0.08 [0.00, 0.16]**	**0.037**	**Word Definitions**	**0.09 [0.01, 0.16]**	**0.024**
Words Memory Delayed	0.02 [-0.05, 0.09]	0.578	Words Memory Delayed	0.06 [-0.01, 0.14]	0.083
Words Memory Immediate	0.05 [-0.02, 0.13]	0.180	Words Memory Immediate	0.06 [-0.01, 0.13]	0.092
**Categorical aMED**			**Categorical MIND**		
**Cognitive scores**	***β*** **(95%CI)**	**p-value**	**Cognitive scores**	***β*** **(95%CI)**	**p-value**
G: Global			G: Global		

T2	0.03 [-0.15, 0.20]	0.776	T2	0.08 [-0.11, 0.26]	0.408
T3	0.07 [-0.11, 0.26]	0.430	T3	0.13 [-0.06, 0.31]	0.172
PC0: Objects memory			PC0: Objects memory		
T2	-0.14 [-0.32, 0.04]	0.138	T2	-0.02 [-0.20, 0.16]	0.808
T3	-0.14 [-0.32, 0.04]	0.138	T3	-0.07 [-0.26, 0.13]	0.495
PC1: Problem solving			PC1: Problem solving		
T2	0.00 [-0.18, 0.18]	0.982	T2	-0.09 [-0.27, 0.09]	0.322
T3	0.09 [-0.09, 0.27]	0.330	T3	0.11 [-0.07, 0.29]	0.224
**PC2: IPS**			PC2: IPS		
T2	**-0.23 [-0.40, -0.06]**	**0.007**	T2	-0.12 [-0.30, 0.06]	0.192
T3	-0.15 [-0.33, 0.03]	0.098	T3	-0.12 [-0.30, 0.06]	0.185
PC3: Words memory			PC3: Words memory		
T2	-0.04 [-0.21, 0.14]	0.675	T2	**0.22 [0.05, 0.40]**	**0.013**
T3	0.07 [-0.12, 0.26]	0.477	T3	0.11 [-0.07, 0.29]	0.243
2D Manipulations			2D Manipulations		
T2	0.02 [-0.15, 0.18]	0.843	T2	-0.03 [-0.22, 0.16]	0.758
T3	-0.09 [-0.28, 0.11]	0.398	T3	-0.01 [-0.19, 0.17]	0.913
Blocks			Blocks		
T2	-0.09 [-0.27, 0.08]	0.297	T2	-0.06 [-0.24, 0.11]	0.482
T3	-0.02 [-0.21, 0.17]	0.813	T3	0.07 [-0.12, 0.25]	0.477
Card Pairs			Card Pairs		
T2	-0.02 [-0.20, 0.16]	0.816	T2	0.05 [-0.14, 0.23]	0.624
T3	-0.03 [-0.21, 0.16]	0.766	T3	0.07 [-0.11, 0.24]	0.460
**Motor Control**			Motor Control		
T2	**-0.19 [-0.36, -0.01]**	**0.038**	T2	-0.04 [-0.21, 0.14]	0.688
T3	-0.15 [-0.33, 0.04]	0.118	T3	-0.06 [-0.25, 0.12]	0.506
Objects Memory Delayed			Objects Memory Delayed		

T2	-0.12 [-0.30, 0.06]	0.209	T2	-0.00 [-0.18, 0.18]	0.966
T3	-0.11 [-0.29, 0.07]	0.238	T3	-0.01 [-0.20, 0.18]	0.921
Objects Memory Immediate			Objects Memory Immediate		
T2	-0.08 [-0.26, 0.10]	0.360	T2	-0.01 [-0.20, 0.17]	0.876
T3	-0.06 [-0.25, 0.13]	0.540	T3	-0.05 [-0.25, 0.14]	0.577
SRT			**SRT**		
T2	-0.07 [-0.25, 0.11]	0.435	T2	-0.14 [-0.32, 0.05]	0.145
T3	-0.17 [-0.36, 0.02]	0.082	T3	**-0.19 [-0.38, -0.01]**	**0.045**
Switching Stroop			Switching Stroop		
T2	-0.11 [-0.29, 0.07]	0.245	T2	-0.12 [-0.30, 0.07]	0.217
T3	0.14 [-0.05, 0.33]	0.160	T3	0.11 [-0.07, 0.30]	0.230
Trail Making			Trail Making		
T2	-0.14 [-0.30, 0.03]	0.103	T2	-0.04 [-0.22, 0.13]	0.623
T3	-0.11 [-0.28, 0.07]	0.242	T3	-0.11 [-0.29, 0.07]	0.243
**Verbal Analogies**			Verbal Analogies		
T2	**0.31 [0.13, 0.48]**	**<0.001**	T2	-0.00 [-0.19, 0.18]	0.984
T3	0.16 [-0.02, 0.34]	0.085	T3	0.10 [-0.08, 0.28]	0.286
**Word Definitions**			**Word Definitions**		
T2	0.01 [-0.16, 0.18]	0.946	T2	0.04 [-0.14, 0.21]	0.663
T3	**0.20 [0.02, 0.38]**	**0.031**	T3	**0.21 [0.03, 0.39]**	**0.022**
Words Memory Delayed			**Words Memory Delayed**		
T2	-0.07 [-0.25, 0.10]	0.412	T2	**0.19 [0.01, 0.37]**	**0.036**
T3	0.03 [-0.15, 0.22]	0.745	T3	0.11 [-0.08, 0.29]	0.257
Words Memory Immediate			**Words Memory Immediate**		
T2	0.12 [-0.05, 0.29]	0.179	T2	**0.18 [0.00, 0.36]**	**0.043**
T3	0.13 [-0.06, 0.32]	0.178	T3	0.08 [-0.10, 0.25]	0.399

Note: Categorical aMED reference group is T1 (lowest tertile); T2=2nd tertile and T3=highest tertile. Bold Test indicates significant results (p<0.05). Abbreviation: aMED=adjusted Mediterranean diet; IPS=Information processing speed; SRT=Simple Reaction Time.

Supplementary Table S3. Sensitivity analysis using Winsorised Deviation from Expected (DfE) scores: associations with aMED and MIND diet scores

Total aMED			Total MIND		
**Cognitive scores**	***β*** **(95%CI)**	**p-value**	**Cognitive scores**	***β*** **(95%CI)**	**p-value**
G: Global	0.04 [-0.03, 0.10]	0.269	G: Global	0.07 [-0.00, 0.14]	0.052
PC0: Objects memory	-0.05 [-0.11, 0.02]	0.183	PC0: Objects memory	0.00 [-0.07, 0.07]	0.909
PC1: Problem solving	0.02 [-0.03, 0.08]	0.378	PC1: Problem solving	0.04 [-0.01, 0.09]	0.151
**PC2: IPS**	**-0.06 [-0.12, -0.01]**	**0.019**	PC2: IPS	-0.03 [-0.09, 0.02]	0.228
PC3: Words memory	0.03 [-0.02, 0.09]	0.237	**PC3: Words memory**	**0.06 [0.01, 0.12]**	**0.032**
2D Manipulations	-0.02 [-0.09, 0.05]	0.558	2D Manipulations	-0.02 [-0.09, 0.04]	0.520
Blocks	-0.03 [-0.11, 0.06]	0.527	Blocks	0.02 [-0.06, 0.10]	0.566
Card Pairs	-0.01 [-0.08, 0.05]	0.685	Card Pairs	0.02 [-0.04, 0.09]	0.471
Motor Control	**-0.10 [-0.20, -0.01]**	**0.030**	Motor Control	-0.06 [-0.16, 0.04]	0.254
Objects Memory Delayed	-0.03 [-0.10, 0.04]	0.404	Objects Memory Delayed	0.03 [-0.05, 0.11]	0.453
Objects Memory Immediate	-0.03 [-0.11, 0.04]	0.388	Objects Memory Immediate	0.00 [-0.08, 0.08]	0.946
**SRT**	**-0.12 [-0.24, -0.00]**	**0.046**	**SRT**	**-0.12 [-0.24, -0.01]**	**0.034**
Switching Stroop	0.05 [-0.03, 0.13]	0.188	Switching Stroop	0.07 [-0.00, 0.15]	0.061
Trail Making	-0.10 [-0.23, 0.02]	0.091	Trail Making	-0.08 [-0.21, 0.06]	0.253
**Verbal Analogies**	**0.07 [0.01, 0.13]**	**0.024**	Verbal Analogies	0.03 [-0.03, 0.09]	0.362
**Word Definitions**	**0.08 [0.02, 0.15]**	**0.017**	**Word Definitions**	**0.09 [0.02, 0.16]**	**0.010**
Words Memory Delayed	0.03 [-0.05, 0.10]	0.505	Words Memory Delayed	0.07 [-0.01, 0.15]	0.074
Words Memory Immediate	0.06 [-0.02, 0.14]	0.152	Words Memory Immediate	0.07 [-0.01, 0.15]	0.071
**Categorical aMED**			**Categorical MIND**		
**Cognitive scores**	***β*** **(95%CI)**	**p-value**	**Cognitive scores**	***β*** **(95%CI)**	**p-value**
G: Global			G: Global		
T2	0.03 [-0.13, 0.20]	0.696	T2	0.09 [-0.08, 0.25]	0.289

T3	0.08 [-0.08, 0.25]	0.326	T3	0.12 [-0.05, 0.29]	0.155
PC0: Objects memory			PC0: Objects memory		
T2	-0.12 [-0.29, 0.05]	0.154	T2	-0.01 [-0.18, 0.16]	0.915
T3	-0.10 [-0.27, 0.06]	0.223	T3	-0.05 [-0.23, 0.12]	0.549
PC1: Problem solving			PC1: Problem solving		
T2	0.01 [-0.12, 0.15]	0.868	T2	-0.08 [-0.22, 0.06]	0.264
T3	0.07 [-0.06, 0.21]	0.285	T3	0.08 [-0.06, 0.21]	0.247
**PC2: IPS**			**PC2: IPS**		
T2	**-0.18 [-0.30, -0.05]**	**0.005**	T2	**-0.13 [-0.26, -0.01]**	**0.046**
T3	-0.11 [-0.24, 0.03]	0.118	T3	-0.12 [-0.25, 0.02]	0.089
PC3: Words memory			**PC3: Words memory**		
T2	-0.01 [-0.15, 0.13]	0.845	T2	**0.19 [0.05, 0.32]**	**0.006**
T3	0.09 [-0.05, 0.24]	0.216	T3	0.10 [-0.04, 0.25]	0.151
2D Manipulations			2D Manipulations		
T2	0.02 [-0.14, 0.17]	0.836	T2	-0.03 [-0.20, 0.13]	0.694
T3	-0.09 [-0.27, 0.08]	0.290	T3	-0.04 [-0.21, 0.12]	0.608
Blocks			Blocks		
T2	-0.09 [-0.28, 0.10]	0.344	T2	-0.07 [-0.25, 0.12]	0.486
T3	-0.02 [-0.22, 0.18]	0.849	T3	0.07 [-0.13, 0.27]	0.502
Card Pairs			Card Pairs		
T2	-0.02 [-0.19, 0.15]	0.832	T2	0.05 [-0.12, 0.22]	0.565
T3	-0.03 [-0.21, 0.14]	0.726	T3	0.05 [-0.12, 0.22]	0.560
Motor Control			Motor Control		
T2	-0.20 [-0.43, 0.02]	0.079	T2	-0.19 [-0.42, 0.03]	0.090
T3	-0.21 [-0.43, 0.02]	0.075	T3	-0.14 [-0.39, 0.11]	0.184
Objects Memory Delayed			Objects Memory Delayed		
T2	-0.11 [-0.29, 0.07]	0.247	T2	0.02 [-0.16, 0.21]	0.804

T3	-0.08 [-0.26, 0.10]	0.388	T3	-0.00 [-0.19, 0.19]	0.990
Objects Memory Immediate			Objects Memory Immediate		
T2	-0.08 [-0.26, 0.10]	0.380	T2	-0.01 [-0.19, 0.17]	0.911
T3	-0.05 [-0.24, 0.14]	0.614	T3	-0.06 [-0.25, 0.14]	0.560
SRT			**SRT**		
T2	-0.11 [-0.39, 0.17]	0.429	**T2**	**-0.29 [-0.56, -0.01]**	**0.039**
T3	-0.22 [-0.51, 0.07]	0.141	**T3**	**-0.36 [-0.64, -0.07]**	**0.016**
Switching Stroop			Switching Stroop		
T2	-0.08 [-0.27, 0.11]	0.399	T2	-0.14 [-0.33, 0.06]	0.161
T3	0.16 [-0.03, 0.36]	0.101	T3	0.12 [-0.07, 0.31]	0.217
Trail Making			Trail Making		
T2	**-0.33 [-0.62, -0.03]**	**0.029**	T2	-0.15 [-0.45, 0.16]	0.356
T3	-0.23 [-0.56, 0.09]	0.163	T3	-0.19 [-0.52, 0.13]	0.245
**Verbal Analogies**			Verbal Analogies		
T2	**0.24 [0.10, 0.38]**	**<0.001**	T2	-0.01 [-0.16, 0.13]	0.851
T3	0.12 [-0.02, 0.27]	0.092	T3	0.07 [-0.08, 0.22]	0.340
**Word Definitions**			**Word Definitions**		
T2	0.03 [-0.13, 0.19]	0.717	T2	0.07 [-0.10, 0.23]	0.417
T3	**0.19 [0.02, 0.35]**	**0.024**	T3	**0.21 [0.04, 0.37]**	**0.016**
Words Memory Delayed			**Words Memory Delayed**		
T2	-0.09 [-0.28, 0.11]	0.383	T2	**0.22 [0.03, 0.40]**	**0.024**
T3	0.06 [-0.13, 0.26]	0.520	T3	0.12 [-0.07, 0.32]	0.219
Words Memory Immediate			**Words Memory Immediate**		
T2	0.12 [-0.06, 0.31]	0.193	T2	**0.19 [0.01, 0.38]**	**0.044**
T3	0.16 [-0.04, 0.37]	0.116	T3	0.10 [-0.09, 0.30]	0.311

Abbreviation: aMED=adjusted Mediterranean diet; IPS=Information processing speed;. SRT=Simple Reaction Time.

Subgroup analyses:

Supplementary Table S4. Examination of associations between aMED and cognition moderated by MS phenotype

Continuous aMED						
**Cognitive diet score**	**Non-progressive**		**Progressive**		**Group difference**	
	***β*(95%CIs)**	***p***	***β*(95%CIs)**	***p***	***β*(95%CIs)**	***p***
**G: Global**	0.01 [-0.08, 0.09]	0.866	0.06 [-0.06, 0.18]	0.332	0.05 (-0.09, 0.20)	0.471
**PC0: Objects memory**	-0.05 [-0.14, 0.04]	0.261	-0.06 [-0.18, 0.05]	0.270	0.00 (-0.15, 0.15)	0.984
Objects Memory Delayed	-0.03 [-0.13, 0.06]	0.502	-0.06 [-0.18, 0.06]	0.334	-0.01 (-0.18, 0.15)	0.873
Objects Memory Immediate	-0.07 [-0.17, 0.03]	0.196	-0.00 [-0.13, 0.12]	0.970	0.07 (-0.09, 0.23)	0.394
**PC1: Problem solving**	-0.02 [-0.08, 0.05]	0.662	0.05 [-0.05, 0.15]	0.293	0.06 (-0.06, 0.18)	0.361
2D Manipulations	-0.04 [-0.13, 0.05]	0.359	-0.00 [-0.12, 0.11]	0.962	0.04 (-0.11, 0.18)	0.621
Blocks	-0.06 [-0.16, 0.05]	0.298	-0.02 [-0.17, 0.12]	0.750	0.03 (-0.14, 0.20)	0.704
Card Pairs	-0.05 [-0.14, 0.04]	0.313	0.02 [-0.09, 0.14]	0.695	0.05 (-0.10, 0.20)	0.514
Switching Stroop	0.01 [-0.08, 0.10]	0.883	0.07 [-0.08, 0.21]	0.384	0.05 (-0.12, 0.22)	0.585
Verbal Analogies	0.07 [-0.00, 0.13]	0.066	0.06 [-0.04, 0.16]	0.229	0.04 (-0.09, 0.16)	0.551
**Word Definitions**	0.05 [-0.03, 0.14]	0.229	**0.14 [0.02, 0.25]**	**0.018**	0.08 -0.06, 0.23)	0.265
**PC2: IPS**	-0.05 [-0.11, 0.01]	0.115	-0.09 [-0.20, 0.03]	0.141	-0.05 (-0.16, 0.07)	0.448
Motor Control	-0.08 [-0.18, 0.03]	0.160	-0.14 [-0.35, 0.06]	0.169	-0.09 (-0.30, 0.12)	0.413
SRT	-0.11 [-0.26, 0.04]	0.148	-0.12 [-0.33, 0.10]	0.299	-0.04 (-0.28, 0.21)	0.779
Trail Making	-0.05 [-0.19, 0.09]	0.457	-0.21 [-0.50, 0.08]	0.163	-0.10 (-0.40, 0.20)	0.497
**PC3: Words memory**	0.04 [-0.03, 0.12]	0.280	0.04 [-0.06, 0.14]	0.435	-0.01 (-0.14, 0.11)	0.853
Words Memory Delayed	0.05 [-0.05, 0.15]	0.339	0.03 [-0.10, 0.16]	0.695	-0.03 (-0.20, 0.14)	0.696
Words Memory Immediate	0.03 [-0.07, 0.14]	0.521	0.09 [-0.06, 0.24]	0.235	0.04 (-0.13, 0.22)	0.985
**Categorical aMED**						
**Cognitive diet score**	**Non-progressive**		**Progressive**		**Group difference**	
	***β*(95%CIs)**	***p***	***β*(95%CIs)**	***p***	***β*(95%CIs)**	***p***
**G: Global**						
T2	-0.04 [-0.31, 0.12]	0.394	0.15 [-0.11, 0.41]	0.258	0.25 (-0.08, 0.58)	0.144
T3	0.04 [-0.16, 0.24]	0.695	0.15 [-0.15, 0.44]	0.332	0.14 (-0.21, 0.50)	0.431

**PC0: Objects memory**						
T2	-0.07 [-0.29, 0.16]	0.565	-0.27 [-0.55, 0.00]	0.065	-0.19 (-0.53, 0.15)	0.279
T3	-0.13 [-0.35, 0.09]	0.257	-0.15 [-0.43, 0.14]	0.319	0.01 (-0.36, 0.38)	0.971
Objects Memory Delayed						
T2	-0.08 [-0.33, 0.16]	0.496	-0.22 [-0.53, 0.08]	0.150	-0.12 (-0.49, 0.26)	0.546
T3	-0.10 [-0.34, 0.13]	0.392	-0.15 [-0.47, 0.17]	0.361	-0.02 (-0.43, 0.38)	0.907
Objects Memory Immediate						
T2	-0.09 [-0.34, 0.15]	0.443	-0.16 [-0.46, 0.13]	0.273	-0.06 (-0.44, 0.32)	0.759
T3	-0.15 [-0.40, 0.11]	0.271	0.03 [-0.27, 0.34]	0.838	0.18 (-0.22, 0.59)	0.376
**PC1: Problem solving**						
T2	-0.11 [-0.29, 0.07]	0.230	0.11 [-0.11, 0.33]	0.319	0.21 (-0.07, 0.48)	0.145
T3	-0.02 [-0.19, 0.15]	0.781	0.13 [-0.11, 0.37]	0.276	0.13 (-0.17, 0.43)	0.395
2D Manipulations						
T2	-0.13 [-0.33, 0.08]	0.220	0.16 [-0.07, 0.40]	0.174	0.28 (-0.06, 0.61)	0.108
T3	-0.18 [-0.41, 0.06]	0.137	0.03 [-0.26, 0.32]	0.838	0.20 (-0.16, 0.57)	0.281
Blocks						
T2	-0.27 [-0.52, 0.04]	0.097	0.08 [-0.23, 0.39]	0.610	0.36 (-0.03, 0.75)	0.073
T3	-0.12 [-0.37, 0.14]	0.379	0.01 [-0.35, 0.38]	0.945	0.12 (-0.30, 0.55)	0.569
Card Pairs						
T2	-0.14 [-0.38, 0.09]	0.237	0.10 [-0.16, 0.37]	0.431	0.23 (-0.12, 0.59)	0.197
T3	-0.11 [-0.35, 0.12]	0.342	0.07 [-0.21, 0.35]	0.629	0.14 (-0.24, 0.53)	0.458
Switching Stroop						
T2	-0.18 [-0.42, 0.05]	0.130	-0.05 [-0.38, 0.28]	0.774	0.11 (-0.28, 0.50)	0.567
T3	0.03 [-0.20, 0.26]	0.809	0.22 [-0.14, 0.58]	0.227	0.16 (-0.26, 0.58)	0.468
**Verbal Analogies**						
T2	**0.26 [0.06, 0.45]**	**0.010**	**0.22 [0.02, 0.41]**	**0.032**	0.06 (-0.23, 0.35)	0.699
T3	0.09 [-0.10, 0.28]	0.374	0.09 [-0.14, 0.32]	0.460	0.06 (-0.26, 0.37)	0.717
**Word Definitions**						
T2	0.06 [-0.16, 0.28]	0.596	-0.00 [-0.27, 0.27]	0.994	-0.06 (-0.40, 0.28)	0.731
T3	0.15 [-0.07, 0.37]	0.178	**0.27 [0.01, 0.53]**	**0.043**	0.13 (-0.24, 0.49)	0.501

**PC2: IPS**						
T2	-0.08 [-0.24, 0.07]	0.299	**-0.32 [-0.57, -0.08]**	**0.008** ^	-0.24 (-0.51, 0.03)	0.083
T3	-0.09 [-0.24, 0.07]	0.260	-0.17 [-0.45, 0.10]	0.219	-0.09 (-0.39, 0.20)	0.526
Motor Control						
T2	-0.04 [-0.32, 0.24]	0.757	-0.43 [-0.90, 0.05]	0.077	-0.38 (-0.89, 0.11)	0.129
T3	-0.15 [-0.41, 0.11]	0.260	-0.36 [-0.86, 0.13]	0.153	-0.23 (-0.76, 0.30)	0.393
SRT						
T2	-0.10 [-0.43, 0.23]	0.563	-0.16 [-0.68, 0.35]	0.536	-0.11 (-0.68, 0.46)	0.709
T3	-0.22 [-0.59, 0.15]	0.252	-0.27 [-0.80, 0.26]	0.316	-0.10 (-0.72, 0.52)	0.748
**Trail Making**						
T2	-0.08 [-0.51, 0.35]	0.719	**-0.88 [-1.46, -0.29]**	**0.003** ^	**-0.73 (-1.42, -0.05)**	**0.036**
T3	-0.20 [-0.59, 0.18]	0.304	-0.35 [-1.10, 0.40]	0.358	-0.03 (-0.77, 0.72)	0.946
**PC3: Words memory**						
T2	-0.10 [-0.28, 0.08]	0.281	0.14 [-0.09, 0.37]	0.244	0.24 (-0.05, 0.53)	0.103
T3	0.06 [-0.14, 0.25]	0.572	0.12 [-0.13, 0.38]	0.328	0.04 (-0.27, 0.35)	0.819
Words Memory Delayed						
T2	-0.12 [-0.37, 0.12]	0.329	0.05 [-0.28, 0.38]	0.771	0.18 (-0.21, 0.57)	0.366
T3	0.04 [-0.22, 0.29]	0.778	0.11 [-0.22, 0.45]	0.501	0.04 (-0.38, 0.47)	0.839
**Words Memory Immediate**						
T2	-0.12 [-0.36, 0.12]	0.326	**0.45 [0.14, 0.75]**	**0.004** ^	**0.57 (0.17, 0.97)**	**0.006**
T3	0.08 [-0.20, 0.36]	0.583	0.21 [-0.15, 0.56]	0.257	0.09 (-0.34, 0.52)	0.687

Note: Categorical aMED reference group is T1 (lowest tertile); T2=2nd tertile and T3=highest tertile. Bold Test indicates significant results (p<0.05). Abbreviation: aMED=adjusted Mediterranean diet; IPS=Information processing speed. SRT=Simple Reaction Time.

Diet and cognitive performance by moderators

Supplementary Table S5. Examination of associations between aMED and cognitive performance moderated by DMT use

**Continuous aMED**						
**Cognitive diet score**	**No DMTs**		**DMTs**		**Group difference**	
	***β*(95%CIs)**	***p***	***β*(95%CIs)**	***p***	***β*(95%CIs)**	***p***
**G: Global**	0.03 [-0.07, 0.13]	0.579	0.03 [-0.06, 0.12]	0.561	-0.00 (-0.14, 0.14)	0.988
**PC0: Objects memory**	-0.03 [-0.14, 0.08]	0.596	-0.08 [-0.17, 0.01]	0.099	-0.04 (-0.18, 0.10)	0.583
Objects Memory Delayed	-0.02 [-0.13, 0.10]	0.743	-0.06 [-0.16, 0.04]	0.235	-0.03 (-0.19, 0.12)	0.667
Objects Memory Immediate	-0.02 [-0.14, 0.10]	0.752	-0.06 [-0.17, 0.04]	0.243	-0.04 (-0.19, 0.12)	0.659
**PC1: Problem solving**	0.03 [-0.06, 0.12]	0.491	0.00 [-0.07, 0.07]	0.950	-0.03 (-0.15, 0.08)	0.571
2D Manipulations	0.03 [-0.07, 0.14]	0.550	-0.07 [-0.16, 0.03]	0.170	-0.11 (-0.25, 0.03)	0.122
Blocks	0.01 [-0.11, 0.14]	0.856	-0.07 [-0.19, 0.04]	0.212	-0.07 (-0.24, 0.09)	0.382
Card Pairs	-0.03 [-0.15, 0.08]	0.569	-0.00 [-0.09, 0.09]	0.967	0.04 (-0.11, 0.19)	0.598
Switching Stroop	-0.01 [-0.13, 0.12]	0.923	0.06 [-0.04, 0.16]	0.245	0.06 (-0.10, 0.22)	0.477
Verbal Analogies	0.07 [-0.02, 0.17]	0.136	0.05 [-0.03, 0.13]	0.264	-0.04 (-0.16, 0.08)	0.542
Word Definitions	0.09 [-0.02, 0.21]	0.114	0.08 [-0.01, 0.17]	0.070	-0.02 (-0.16, 0.12)	0.798
**PC2: IPS**	-0.06 [-0.15, 0.03]	0.198	-0.06 [-0.14, 0.01]	0.091	0.01 (-0.11, 0.12)	0.975
Motor Control	-0.07 [-0.23, 0.09]	0.394	-0.12 [-0.24, 0.01]	0.061	-0.05 (-0.25, 0.16)	0.657
SRT	-0.03 [-0.21, 0.15]	0.758	-0.08 [-0.34, -0.01]	0.117	-0.13 (-0.37, 0.11)	0.286
Trail Making	-0.14 [-0.37, 0.09]	0.222	-0.09 [-0.26, 0.09]	0.327	0.07 (-0.22, 0.36)	0.638
**PC3: Words memory**	-0.01 [-0.11, 0.09]	0.790	0.08 [-0.01, 0.15]	0.059	0.10 (-0.02, 0.23)	0.101
Words Memory Delayed	-0.03 [-0.16, 0.10]	0.649	0.09 [-0.02, 0.20]	0.095	0.13 (-0.03, 0.30)	0.121
Words Memory Immediate	0.04 [-0.11, 0.18]	0.624	0.07 [-0.03, 0.18]	0.170	0.05 (-0.12, 0.22)	0.581
**Categorical aMED**						
**Cognitive diet score**	**No DMTs**		**DMTs**		**Group difference**	
	***β*(95%CIs)**	***p***	***β*(95%CIs)**	***p***	***β*(95%CIs)**	***p***
**G: Global**						
T2	0.14 [-0.10, 0.39]	0.243	-0.11 [-0.34, 0.12]	0.362	-0.39 (-0.61, 0.03)	0.071
T3	0.03 [-0.24, 0.29]	0.838	0.06 [-0.15, 0.28]	0.564	-0.01 (-0.37, 0.34)	0.951
**PC0: Objects memory**						

T2	-0.13 [-0.38, 0.12]	0.297	-0.17 [-0.42, 0.07]	0.156	-0.06 (-0.39, 0.28)	0.733
T3	-0.09 [-0.36, 0.18]	0.517	-0.15 [-0.39, 0.08]	0.189	-0.07 (-0.44, 0.31)	0.731
Objects Memory Delayed						
T2	-0.09 [-0.36, 0.19]	0.540	-0.20 [-0.47, 0.06]	0.136	-0.13 (-0.50, 0.23)	0.474
T3	-0.09 [-0.39, 0.21]	0.571	-0.13 [-0.38, 0.12]	0.299	-0.04 (-0.45, 0.36)	0.832
Objects Memory Immediate						
T2	-0.06 [-0.33, 0.21]	0.654	-0.16 [-0.43, 0.10]	0.228	-0.13 (-0.50, 0.24)	0.485
T3	-0.05 [-0.35, 0.25]	0.739	-0.10 [-0.36, 0.17]	0.471	-0.09 (-0.50, 0.32)	0.678
**PC1: Problem solving**						
T2	0.09 [-0.11, 0.29]	0.394	-0.13 [-0.31, 0.06]	0.187	-0.25 (-0.53, 0.02)	0.069
T3	0.06 [-0.16, 0.28]	0.587	0.01 [-0.17, 0.19]	0.928	-0.10 (-0.41, 0.20)	0.501
2D Manipulations						
T2	0.19 [-0.02, 0.41]	0.080	-0.20 [-0.43, 0.02]	0.076	**-0.44 (-0.81, -0.08)**	**0.016**
T3	0.08 [-0.19, 0.34]	0.575	-0.23 [-0.48, 0.02]	0.068	-0.37 (-0.76, 0.02)	0.065
Blocks						
T2	-0.03 [-0.31, 0.25]	0.818	-0.24 [-0.51, 0.03]	0.081	-0.23 (-0.62, 0.15)	0.235
T3	0.07 [-0.26, 0.39]	0.691	-0.17 [-0.45, 0.11]	0.234	-0.20 (-0.63, 0.23)	0.356
Card Pairs						
T2	0.07 [-0.18, 0.32]	0.598	-0.11 [-0.37, 0.14]	0.386	-0.16 (-0.51, 0.19)	0.369
T3	-0.12 [-0.40, 0.17]	0.425	0.01 [-0.23, 0.24]	0.955	0.15 (-0.24, 0.53)	0.448
Switching Stroop						
T2	-0.04 [-0.32, 0.25]	0.799	-0.22 [-0.48, 0.05]	0.108	-0.26 (-0.64, 0.11)	0.168
T3	0.01 [-0.31, 0.32]	0.965	0.16 [-0.10, 0.41]	0.222	0.03 (-0.39, 0.44)	0.905
**Verbal Analogies**						
T2	**0.26 [0.06, 0.45]**	**0.010**	**0.24 [0.03, 0.45]**	**0.024**	-0.05 (-0.33, 0.23)	0.731
T3	0.12 [-0.11, 0.34]	0.299	0.08 [-0.12, 0.28]	0.421	-0.06 (-0.36, 0.25)	0.725
**Word Definitions**						
T2	-0.02 [-0.28, 0.24]	0.873	0.09 [-0.13, 0.31]	0.430	0.07 (-0.25, 0.40)	0.655
T3	0.17 [-0.09, 0.43]	0.201	0.22 [-0.01, 0.45]	0.059	0.03 (-0.33, 0.38)	0.889
**PC2: IPS**						

T2	**-0.25 [-0.45, -0.05]**	**0.015**	-0.12 [-0.30, 0.06]	0.184	0.15 (-0.11, 0.41)	0.257
T3	-0.12 [-0.35, 0.11]	0.315	-0.13 [-0.31, 0.05]	0.150	0.03 (-0.27, 0.32)	0.863
Motor Control						
T2	-0.29 [-0.67, 0.09]	0.130	-0.12 [-0.45, 0.21]	0.474	0.24 (-0.24, 0.73)	0.324
T3	-0.20 [-0.62, 0.22]	0.349	-0.26 [-0.56, 0.03]	0.076	0.02 (-0.52, 0.56)	0.953
**SRT**						
T2	-0.02 [-0.46, 0.42]	0.926	-0.25 [-0.62, 0.13]	0.194	-0.15 (-0.71, 0.40)	0.591
T3	-0.04 [-0.53, 0.46]	0.884	**-0.41 [-0.80, -0.02]**	**0.040**	-0.32 (-0.94, 0.29)	0.304
**Trail Making**						
T2	**-0.66 [-1.20, -0.12]**	**0.017** ^	-0.10 [-0.54, 0.34]	0.665	0.52 [-0.12, 1.16]	0.112
T3	-0.27 [-0.89, 0.36]	0.403	-0.23 [-0.67, 0.22]	0.317	0.17 (-0.59, 0.92)	0.668
**PC3: Words memory**						
T2	0.09 [-0.11, 0.28]	0.392	-0.03 [-0.23, 0.18]	0.803	-0.10 (-0.38, 0.18)	0.489
T3	-0.07 [-0.33, 0.19]	0.607	0.18 [-0.01, 0.37]	0.070	0.26 (-0.05, 0.57)	0.095
Words Memory Delayed						
T2	0.03 [-0.25, 0.31]	0.826	-0.07 [-0.34, 0.21]	0.634	-0.05 (-0.43, 0.33)	0.808
T3	-0.12 [-0.44, 0.20]	0.455	0.18 [-0.08, 0.44]	0.177	0.31 (-0.10, 0.73)	0.139
**Words Memory Immediate**						
T2	**0.33 [0.07, 0.60]**	**0.012** ^	-0.10 [-0.39, 0.19]	0.508	**-0.43 (-0.82, -0.05)**	**0.029**
T3	0.08 [-0.30, 0.46]	0.678	0.19 [-0.08, 0.45]	0.162	0.18 (-0.24, 0.61)	0.459

Note: Categorical aMED reference group is T1 (lowest tertile); T2=2nd tertile and T3=highest tertile. Bold Test indicates significant results (*p*<0.05). Abbreviation: aMED=adjusted Mediterranean diet; IPS=Information processing speed. SRT=Simple Reaction Time.

1.

2.

Supplementary Table S6. Examination of associations between MIND diet scores and cognition moderated by MS phenotype

Continuous MIND						
**Cognitive score**	**Non-progressive**		**Progressive**		**Group difference**	
	***β*(95%CIs)**	***p***	***β*(95%CIs)**	***p***	***β*(95%CIs)**	***p***
**G: Global**	0.04 [-0.04, 0.13]	0.346	0.10 [-0.02, 0.24]	0.093	0.07 (-0.07, 0.21)	0.318
**PC0: Objects memory**	-0.02 [-0.11, 0.08]	0.707	0.07 [-0.05, 0.18]	0.235	0.08 (-0.07, 0.23)	0.301
Objects Memory Delayed	-0.00 [-0.10, 0.10]	0.981	0.11 [-0.01, 0.23]	0.086	0.09 (-0.07, 0.25)	0.264
Objects Memory Immediate	-0.02 [-0.13, 0.09]	0.771	0.07 [-0.05, 0.20]	0.253	0.08 (-0.08, 0.25)	0.308
**PC1: Problem solving**	0.03 [-0.04, 0.09]	0.411	0.06 [-0.04, 0.16]	0.218	0.02 (-0.10, 0.14)	0.771
2D Manipulations	-0.02 [-0.10, 0.07]	0.709	0.00 [-0.10, 0.11]	0.960	-0.01 (-0.15, 0.14)	0.939
Blocks	0.01 [-0.09, 0.11]	0.874	0.03 [-0.11, 0.17]	0.690	0.02 (-0.15, 0.18)	0.841
Card Pairs	-0.01 [-0.09, 0.08]	0.867	0.08 [-0.03, 0.19]	0.165	0.08 (-0.07, 0.23)	0.267
Switching Stroop	0.06 [-0.03, 0.15]	0.213	0.07 [-0.06, 0.21]	0.296	-0.00 (-0.17, 0.16)	0.968
Verbal Analogies	0.04 [-0.04, 0.11]	0.350	0.03 [-0.07, 0.12]	0.584	-0.03 (-0.16, 0.10)	0.642
**Word Definitions**	0.04 [-0.05, 0.13]	0.406	**0.15 [0.05, 0.25]**	**0.004** ^	0.13 (-0.02, 0.27)	0.079
**PC2: IPS**	-0.04 [-0.11, 0.02]	0.182	-0.04 [-0.15, 0.07]	0.501	0.02 (-0.10, 0.14)	0.727
Motor Control	-0.06 [-0.17, 0.05]	0.300	-0.06 [-0.27, 0.16]	0.599	0.02 (-0.20, 0.23)	0.888
SRT	-0.11 [-0.24, 0.03]	0.117	-0.13 [-0.33, 0.07]	0.200	-0.01 (-0.25, 0.24)	0.962
Trail Making	-0.11 [-0.26, 0.04]	0.138	-0.07 [-0.35, 0.21]	0.610	0.07 (-0.23, 0.37)	0.642
**PC3: Words memory**	0.03 [-0.04, 0.10]	0.386	**0.11 [0.02, 0.20]**	**0.021**	0.09 (-0.03, 0.21)	0.167
**Words Memory Delayed**	0.05 [-0.05, 0.14]	0.334	**0.13 [0.01, 0.25]**	**0.042**	0.09 (-0.08, 0.26)	0.297
Words Memory Immediate	0.02 [-0.08, 0.13]	0.644	0.15 [-0.01, 0.28]	0.057	0.14 (-0.03, 0.30)	0.109
**Categorical MIND**						
**Cognitive score**	**Non-progressive**		**Progressive**		**Group difference**	
	***β*(95%CIs)**	***p***	***β*(95%CIs)**	***p***	***β*(95%CIs)**	***p***
**G: Global**						
T2	0.02 [-0.19, 0.23]	0.839	**0.28 [0.01, 0.54]**	**0.041**	0.30 (-0.04, 0.63)	0.079
T3	0.09 [-0.13, 0.31]	0.424	0.21 [-0.04, 0.56]	0.127	0.20 (-0.14, 0.53)	0.262
PC0: Objects memory						

T2	-0.07 [-0.30, 0.16]	0.549	0.11 [-0.16, 0.38]	0.428	0.20 (-0.15, 0.55)	0.341
T3	-0.12 [-0.36, 0.12]	0.330	0.15 [-0.13, 0.43]	0.303	0.27 (-.08, 0.63)	0.166
Objects Memory Delayed						
T2	-0.06 [-0.31, 0.18]	0.610	0.21 [-0.10, 0.51]	0.180	0.29 (-0.09, 0.67)	0.139
T3	-0.09 [-0.35, 0.17]	0.502	0.25 [-0.06, 0.55]	0.089	0.34 (-0.05, 0.73)	0.089
Objects Memory Immediate						
T2	-0.03 [-0.28, 0.22]	0.805	0.09 [-0.20, 0.38]	0.522	0.14 (-0.25, 0.52)	0.491
T3	-0.08 [-0.35, 0.19]	0.550	0.12 [-0.18, 0.42]	0.442	0.20 (-0.19, 0.59)	0.321
PC1: Problem solving						
T2	-0.08 [-0.27, 0.10]	0.368	0.06 [-0.17, 0.28]	0.627	0.10 (-0.19, 0.38)	0.388
T3	0.09 [-0.09, 0.27]	0.313	0.10 [-0.13, 0.32]	0.407	-0.06 (-0.34, 0.23)	0.699
2D Manipulations						
T2	-0.02 [-0.24, 0.20]	0.845	0.16 [-0.12, 0.44]	0.275	0.12 (-0.22, 0.47)	0.425
T3	0.01 [-0.22, 0.24]	0.924	0.01 [-0.24, 0.26]	0.941	-0.03 (-0.38, 0.32)	0.868
Blocks						
T2	-0.15 [-0.39, 0.09]	0.225	0.17 [-0.15, 0.48]	0.300	0.29 (-0.11, 0.69)	0.155
T3	0.09 [-0.17, 0.34]	0.497	-0.02 [-0.36, 0.32]	0.923	-0.14 (-0.54, 0.26)	0.495
Card Pairs						
T2	-0.05 [-0.29, 0.20]	0.693	0.19 [-0.08, 0.46]	0.161	0.20 (-0.16, 0.56)	0.283
T3	-0.02 [-0.24, 0.21]	0.882	0.21 [-0.05, 0.47]	0.112	0.19 (-0.17, 0.56)	0.296
Switching Stroop						
T2	-0.18 [-0.42, 0.05]	0.131	0.05 [-0.29, 0.38]	0.781	0.22 (-0.17, 0.62)	0.263
T3	0.12 [-0.12, 0.36]	0.342	0.12 [-0.21, 0.45]	0.490	-0.04 (-0.44, 0.36)	0.838
Verbal Analogies						
T2	0.03 [-0.18, 0.23]	0.783	-0.00 [-0.21, 0.21]	0.998	-0.08 (-0.38, 0.22)	0.605
T3	0.08 [-0.12, 0.28]	0.423	0.10 [-0.11, 0.31]	0.358	-0.05 (-0.35, 0.26)	0.757
**Word Definitions**						
T2	-0.02 [-0.24, 0.19]	0.824	0.22 [-0.06, 0.50]	0.119	0.24 (-0.10, 0.59)	0.171
T3	0.11 [-0.12, 0.34]	0.334	**0.41 [0.17, 0.66]**	**0.005** ^	0.25 (-0.10, 0.60)	0.162
**PC2: IPS**						

T2	-0.10 [-0.25, 0.06]	0.239	**-0.24 [-0.48, -0.00]**	**0.049**	-0.16 (-0.44, 0.12)	0.254
T3	-0.15 [-0.31, 0.01]	0.065	-0.15 [-0.41, 0.11]	0.260	0.01 (-0.28, 0.29)	0.841
**Motor Control**						
T2	0.00 [-0.28, 0.29]	0.976	**-0.51 [-0.95, -0.08]**	**0.020**	-0.55 (-1.06, -0.05)	0.085
T3	-0.14 [-0.41, 0.13]	0.308	-0.22 [-0.72, 0.29]	0.403	-0.13 (-0.65, 0.38)	0.611
SRT						
T2	-0.16 [-0.49, 0.18]	0.367	-0.47 [-0.96, 0.03]	0.066	-0.37 (-0.94, 0.20)	0.204
T3	-0.23 [-0.60, 0.15]	0.231	-0.45 [-0.96, 0.05]	0.079	-0.06 (-0.63, 0.52)	0.851
Trail Making						
T2	-0.32 [-0.74, 0.10]	0.133	-0.27 [-0.89, 0.35]	0.394	0.11 (-0.60, 0.82)	0.765
T3	-0.25 [-0.61, 0.12]	0.184	-0.12 [-0.76, 0.52]	0.713	0.36 (-0.36, 1.08)	0.323
**PC3: Words memory**						
T2	0.15 [-0.04, 0.33]	0.118	**0.32 [0.10, 0.53]**	**0.004** ^	0.16 (-0.13, 0.46)	0.268
T3	0.04 [-0.14, 0.23]	0.643	**0.25 [0.02, 0.47]**	**0.030**	0.26 (-0.04, 0.56)	0.084
**Words Memory Delayed**						
T2	-0.16 [-0.40, 0.46]	0.188	**0.36 [0.05, 0.67]**	**0.024**	0.13 (-0.27, 0.53)	0.521
T3	0.09 [-0.16, 0.35]	0.469	0.29 [-0.03, 0.60]	0.072	0.26 (-0.14, 0.67)	0.201
**Words Memory Immediate**						
T2	0.09 [-0.16, 0.34]	0.488	**0.42 [0.11, 0.73]**	**0.005** ^	**0.42 [0.02, 0.83]**	**0.041**
T3	-0.02 [-0.28, 0.23]	0.864	0.30 [-0.00, 0.66]	0.053	0.36 [-0.05, 0.78]	0.085

Note: Categorical MIND reference group is T1 (lowest tertile); T2=2nd tertile and T3=highest tertile. Bold Test indicates significant results (p<0.05). Models were adjusted for total energy intake, disease duration and DMT use. Abbreviation: MIND=Mediterranean-DASH Intervention for Neurodegenerative Delay; IPS=information processing speed; SRT=Simple Reaction Time.

3.

Diet and cognitive performance by moderators

Supplementary Table S7. Subgroup analyses: Associations between MIND and cognitive performance between DMT use vs no DMT use.

Continuous MIND						
Cognitive diet score	**No DMTs**		**DMTs**		**Group difference**	
	***β*(95%CIs)**	***p***	***β*(95%CIs)**	***p***	***β*(95%CIs)**	***p***
**G: Global**	**0.22 [0.05, 0.40]**	**0.014**	**-0.02 [-0.11, 0.08]**	**0.735**	**-0.25 (-0.44, -0.06)**	**0.009**
PC0: Object memory	0.10 [-0.11, 0.31]	0.333	-0.06 [-0.16, 0.05]	0.281	-0.15 (-0.35, 0.05)	0.131
Objects Memory Delayed	0.14 [-0.09, 0.37]	0.240	-0.05 [-0.16, 0.06]	0.410	-0.19 (-0.41, 0.03)	0.090
Objects Memory Immediate	0.17 [-0.06, 0.39]	0.150	-0.08 [-0.19, 0.04]	0.200	**-0.24 (-0.46, -0.01)**	**0.040**
**PC1: Problem solving**	**0.15 [0.02, 0.27]**	**0.021**	-0.00 [-0.07, 0.07]	0.930	-0.15 (-0.31, 0.00)	0.060
2D Manipulations	0.13 [-0.03, 0.28]	0.110	-0.06 [-0.15, 0.03]	0.210	**-0.21 (-0.41, -0.00)**	**0.050**
**Blocks**	**0.21 [0.05, 0.37]**	**0.011**	-0.06 [-0.17, 0.06]	0.320	**-0.26 (-0.48, -0.05)**	**0.020**
Card Pairs	0.01 [-0.16, 0.18]	0.872	0.00 [-0.10, 0.10]	1.001	-0.03 (-0.24, 0.18)	0.780
Switching Stroop	0.15 [-0.04, 0.35]	0.121	0.06 [-0.05, 0.17]	0.260	-0.08 (-0.29, 0.14)	0.480
Verbal Analogies	0.04 [-0.11, 0.19]	0.601	0.04 [-0.05, 0.13]	0.420	-0.03 (-0.21, 0.15)	0.760
**Word Definitions**	**0.22 [0.02, 0.43]**	**0.028**	-0.01 [-0.11, 0.08]	0.760	**-0.22 (-0.42, -0.03)**	**0.030**
PC2: IPS	-0.11 [-0.27, 0.06]	0.201	-0.03 [-0.10, 0.04]	0.440	0.09 (-0.04, 0.23)	0.210
Motor Control	-0.09 [-0.36, 0.18]	0.511	-0.07 [-0.19, 0.06]	0.311	0.03 (-0.21, 0.27)	0.810
SRT	-0.33 [-0.68, 0.01]	0.059	-0.08 [-0.24, 0.08]	0.321	0.22 (-0.08, 0.53)	0.150
**Trail Making**	**-0.32 [-0.61, -0.04]**	**0.025**	-0.01 [-0.18, 0.15]	0.861	0.34 (-0.01, 0.69)	0.051
PC3: Words memory	0.08 [-0.06, 0.21]	0.281	-0.00 [-0.08, 0.08]	0.960	-0.10 (-0.27, 0.06)	0.230
Words Memory Delayed	0.06 [-0.11, 0.24]	0.492	0.01 [-0.09, 0.12]	0.800	-0.08 (-0.31, 0.14)	0.450
Words Memory Immediate	0.21 [-0.02, 0.43]	0.068	-0.05 [-0.16, 0.06]	0.342	**-0.29 (-0.52, -0.05)**	**0.020**

Categorical MIND						
Cognitive diet score	No DMTs		DMTs		Group difference	
	β(95%CIs)	*p*	*β*(95%CIs)	*p*	*β*(95%CIs)	*p*
G: Global						
T2	0.39 [-0.05, 0.83]	0.080	-0.14 [-0.38, 0.09]	0.237	**-0.58 (-1.02, -0.13)**	**0.011**
T3	0.40 [-0.04, 0.84]	0.075	-0.03 [-0.27, 0.21]	0.815	-0.48 (-0.97, 0.01)	0.054
PC0: Objects memory						

T2	0.20 [-0.21, 0.60]	0.345	-0.13 [-0.39, 0.13]	0.330	-0.35 (-0.82, 0.12)	0.151
T3	0.02 [-0.51, 0.54]	0.954	-0.13 [-0.40, 0.14]	0.336	-0.16 (-0.67, 0.36)	0.553
Objects Memory Delayed						
T2	0.20 [-0.26, 0.67]	0.392	-0.13 [-0.41, 0.14]	0.344	-0.38 (-0.90, 0.13)	0.144
T3	0.03 [-0.54, 0.61]	0.911	-0.10 [-0.39, 0.18]	0.479	-0.18 (-0.75, 0.38)	0.530
Objects Memory Immediate						
T2	0.40 [-0.05, 0.86]	0.084	-0.18 [-0.47, 0.10]	0.209	**-0.60 (-1.11, -0.07)**	**0.027**
T3	0.27 [-0.30, 0.83]	0.355	-0.18 [-0.47, 0.11]	0.229	-0.43 (-1.01, 0.14)	0.141
PC1: Problem solving						
T2	0.17 [-0.19, 0.52]	0.360	-0.19 [-0.39, 0.01]	0.059	-0.36 (-0.74, 0.01)	0.058
T3	0.24 [-0.14, 0.62]	0.221	0.05 [-0.13, 0.24]	0.582	-0.19 (-0.60, 0.22)	0.356
2D Manipulations						
T2	0.29 [-0.13, 0.71]	0.170	-0.19 [-0.45, 0.06]	0.140	-0.50 (-0.98, -0.02)	0.042
T3	0.23 [-0.15, 0.61]	0.242	-0.08 [-0.36, 0.19]	0.545	-0.34 (-0.86, 0.19)	0.211
Blocks						
T2	0.07 [-0.33, 0.46]	0.744	-0.27 [-0.55, 0.01]	0.061	-0.33 (-0.85, 0.18)	0.204
T3	0.39 [-0.10, 0.88]	0.121	-0.00 [-0.29, 0.28]	0.980	-0.39 (-0.95, 0.18)	0.177
Card Pairs						
T2	-0.16 [-0.60, 0.28]	0.486	0.03 [-0.25, 0.31]	0.848	0.15 (-0.35, 0.65)	0.556
T3	-0.09 [-0.56, 0.38]	0.703	0.03 [-0.22, 0.29]	0.786	0.08 (-0.47, 0.63)	0.781
Switching Stroop						
T2	0.13 [-0.31, 0.58]	0.558	-0.28 [-0.57, 0.01]	p=0.059	-0.37 (-0.88, 0.13)	0.145
T3	0.17 [-0.37, 0.70]	0.540	0.14 [-0.14, 0.41]	0.326	-0.03 (-0.58, 0.52)	0.902
Verbal Analogies						
T2	0.13 [-0.28, 0.53]	0.532	-0.02 [-0.25, 0.22]	0.888	-0.17 (-0.60, 0.26)	0.433
T3	0.13 [-0.31, 0.56]	0.567	0.07 [-0.15, 0.30]	0.527	-0.03 (-0.50, 0.44)	0.906
Word Definitions						
T2	0.21 [-0.26, 0.69]	0.380	-0.10 [-0.33, 0.13]	0.396	-0.26 (-0.72, 0.20)	0.265
T3	0.40 [-0.10, 0.90]	0.118	0.02 [-0.23, 0.27]	0.890	-0.38 (-0.89, 0.12)	0.137
**PC2: IPS**						

T2	-0.34 [-0.69, 0.02]	0.063	0.00 [-0.17, 0.18]	0.975	**0.35 (0.02, 0.68)**	**0.036**
**T3**	**-0.38 [-0.72, -0.04]**	**0.027**	-0.05 [-0.23, 0.13]	0.558	0.34 (-0.02, 0.70)	0.067
Motor Control						
T2	-0.19 [-0.72, 0.35]	0.493	0.05 [-0.29, 0.38]	0.776	0.24 (-0.33, 0.81)	0.413
T3	-0.32 [-0.88, 0.24]	0.266	-0.07 [-0.37, 0.24]	0.667	0.25 (-0.38, 0.88)	0.431
**SRT**						
T2	-0.69 [-1.46, 0.07]	0.075	0.01 [-0.36, 0.38]	0.965	0.64 (-0.07, 1.35)	0.079
**T3**	**-1.08 [-1.89, -0.27]**	**0.009**	-0.17 [-0.57, 0.23]	0.397	**0.81 (0.03, 1.60)**	**0.042**
**Trail Making**						
**T2**	**-1.18 [-2.18, -0.18]**	**0.021**	0.10 [-0.25, 0.46]	0.578	**1.35 (0.55, 2.15)**	**0.001**
**T3**	**-1.07 [-1.99, -0.16]**	**0.022**	-0.11 [-0.50, 0.27]	0.560	**1.03 (0.14, 1.91)**	**0.023**
PC3: Words memory						
T2	0.07 [-0.26, 0.40]	0.689	0.09 [-0.13, 0.31]	0.406	-0.04 (-0.43, 0.36)	0.854
T3	0.15 [-0.18, 0.49]	0.374	-0.06 [-0.27, 0.15]	0.570	-0.31 (-0.74, 0.12)	0.161
Words Memory Delayed						
T2	0.07 [-0.40, 0.53]	0.775	0.19 [-0.10, 0.48]	0.206	0.12 (-0.40, 0.64)	0.658
T3	0.21 [-0.25, 0.67]	0.366	0.00 [-0.29, 0.30]	0.979	-0.21 (-0.79, 0.36)	0.461
**Words Memory Immediate**						
**T2**	**0.52 [0.02, 1.01]**	**0.040**	-0.12 [-0.42, 0.18]	0.424	**-0.62 (-1.17, -0.07)**	**0.028**
**T3**	**0.54 [0.04, 1.04]**	**0.035**	-0.24 [-0.52, 0.05]	0.101	**-0.77 (-1.37, -0.16)**	**0.015**

Note: Categorical aMED reference group is T1 (lowest tertile); T2=2nd tertile and T3=highest tertile. Bold Test indicates significant results (p<0.05). Abbreviation: MIND=Mediterranean-DASH Intervention for Neurodegenerative Delay; IPS=Information processing speed. SRT=Simple Reaction Time.

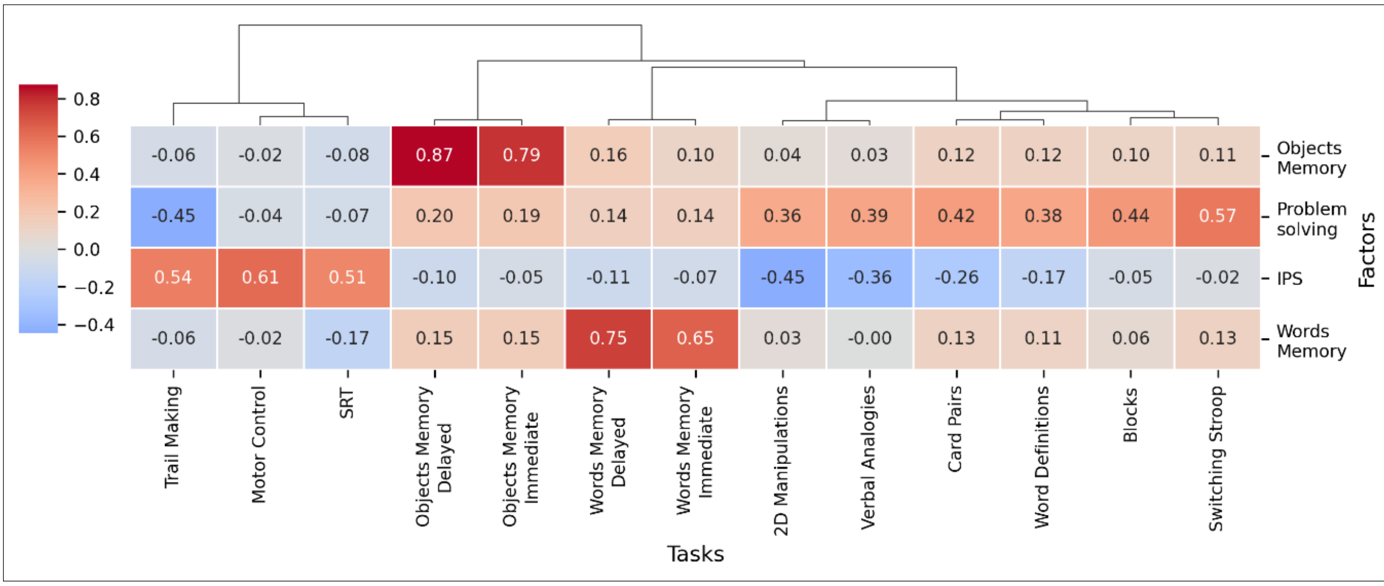

Supplementary Figure S1. Factor analysis of primary cognitive tasks identifying global and domain-specific components

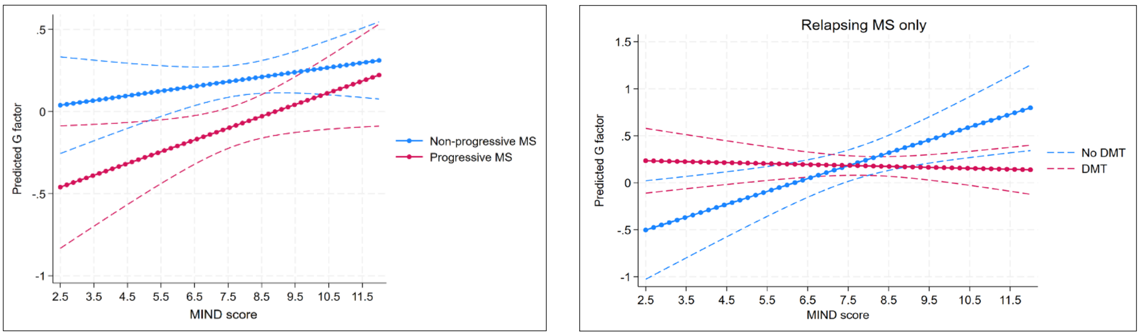

Supplementary Figure S2. Adjusted global cognitive performance (G factor) across MIND diet scores, stratified by MS phenotype (left) and DMT use in relapsing individuals (right).

Note:

Left panel: As MIND scores increase, predicted overall cognition (G factor) increases in both groups. The lines appear to draw closer together at higher scores, indicating smaller differences in overall cognition between progressive and relapsing MS. However, the MIND × MS phenotype interaction was not statistically significant ($p > 0.05$).

Right panel: In relapsing MS, not using a DMT, MIND score was associated with higher overall cognition (G factor), whereas the association is near-zero among DMT users; p for interaction (difference in slopes)=0.009. Models adjust for MS phenotype, disease duration, and total energy intake.
